# Research Progress on NINJ1-Mediated Plasma Membrane Rupture Regulation of Pathogen Infection Process

**DOI:** 10.3390/ijms27104395

**Published:** 2026-05-14

**Authors:** Houjun Ran, Xiaoquan Wang, Kaituo Liu, Xiufan Liu

**Affiliations:** 1 Joint International Research Laboratory of Agriculture and Agri-Product Safety, The Ministry of Education of China, Yangzhou University, Yangzhou 225009, China; 221404414@stu.yzu.edu.cn (H.R.); wxq@yzu.edu.cn (X.W.); xfliu@yzu.edu.cn (X.L.); 2 Key Laboratory of Avian Bioproducts Development, College of Veterinary Medicine, Yangzhou University, Yangzhou 225009, China

**Keywords:** plasma membrane rupture (PMR), lytic cell, death pathogen infection, NINJ1, disease treatment

## Abstract

Pathological events in a wide range of diseases, from severe infections to sterile inflammatory disorders. PMR serves as the terminal step that releases large quantities of damage-associated molecular patterns (DAMPs)—intracellular molecules that act as danger signals once outside the cell. These DAMPs can trigger strong inflammatory responses, and in many cases, may precipitate a cytokine storm, a hyperactive immune reaction that often amplifies tissue injury beyond the initial insult. For decades, scientists generally believed that PMR resulted from membrane pore-forming cell death, such as pyroptosis or necroptosis, which causes osmotic imbalance and passive membrane swelling. However, in recent years, it was discovered that Nerve Injury-Induced Protein 1 (NINJ1) mediates PMR through active oligomerization. This review first uses pathogen infection as a classical model to explore how multiple cell death pathways converge on plasma membrane rupture (PMR). Subsequently, we elaborate on the structure and function of NINJ1 as a core executor of PMR. Finally, we broaden our perspective from infection to other non-infectious but equally PMR-driven major diseases, and systematically evaluate the commonalities and prospects of NINJ1-targeted therapeutic strategies across different pathological scenarios. It is hoped that this will provide new insights for future researchers in this field.

## 1. Introduction

Nerve Injury-Induced Protein 1 (NINJ1) is a protein with a molecular weight of 16.3 kDa, initially identified as a novel adhesion factor in the central nervous system, involved in nerve repair and promoting axonal growth [1]. Beyond its role in nerve regeneration, NINJ1 was characterized as mediating cell-to-cell interactions, particularly facilitating the transendothelial transport of monocytes and macrophages during neuroinflammation [2]. The protein contains an evolutionarily conserved extracellular domain critical for its oligomerization function [3]. Subsequent studies have shown that it is expressed in most organs, with high expression in the skin and ileum, moderate expression in several other organs, and lower expression levels in the brain [4]. This broad expression profile has shifted the research focus toward the physiological and pathological roles of NINJ1 outside the nervous system.

Prior to 2021, NINJ1 was recognized for its involvement in inflammation and tissue remodeling, where it enhanced the entry, migration, and activity of leukocytes such as monocytes and macrophages [2]. Additionally, NINJ1 was found to contribute to Toll-like receptor 4 (TLR4) signaling and systemic inflammation by modulating p38 phosphorylation and activator protein-1 activation [5]. The protein was also implicated in cellular senescence and p53-dependent cell survival, forming a feedback loop with p53 by regulating p53 mRNA translation [6]. It was not until 2021 that Kayagaki et al. first revealed that NINJ1 can mediate plasma membrane rupture during the process of lytic cell death [3], a discovery that has drawn widespread attention.

Lytic cell death caused by pathogen infection is a crucial event in the development of various diseases, especially the final plasma membrane rupture (PMR) step, which results in the release of intracellular contents and triggers a strong systemic inflammatory response [3]. Pyroptosis and necroptosis, two archetypal forms of lytic cell death, initiate the loss of membrane integrity through the pore-forming activities of their respective executioner proteins (GSDMs and MLKL), which permit the transit of ions, small molecules, and water. However, pore formation alone proves insufficient to achieve the complete membrane disintegration necessary for the release of large DAMPs and the ensuing amplification of inflammation. The prevailing paradigm historically posited that this terminal rupture represented a passive phenomenon driven by osmotic dysregulation and uncontrolled cellular swelling. The recent discovery of Nerve Injury-Induced Protein 1 (NINJ1) as an active executor of PMR—functioning through regulated oligomerization—has fundamentally transformed this conceptual framework. In this review, the role of PMR and the activation mechanisms of NINJ1 are first examined within the context of pathogen infection, which provides a robust theoretical foundation for dissecting lytic cell death. Building upon this framework, the scope is extended to encompass other PMR-driven, non-infectious pathological conditions, with systematic evaluation of the therapeutic potential of NINJ1 targeting across a diverse spectrum of inflammatory diseases.

## 2. The Role of Plasma Membrane Rupture in Pathogen Infection Process

### 2.1. Pathogen Infection Induces Multiple Forms of Cell Death

Cell death is a fundamental process in all organisms and plays a crucial role in maintaining homeostasis, development, and disease progression. In nature, cell death can be triggered by various internal and external factors, such as pathogen infections, physical damage, and toxic compounds, among which pathogen infection represents one of the most important pathways for inducing cell death and can induce multiple types of cell death (Table 1). Influenza A virus (IAV) is a single-stranded negative-sense RNA virus belonging to the Orthomyxoviridae family. Due to its high infectivity and variability, it poses a significant threat to public health. Research on its role in cell death is extensive. Current studies show that IAV-induced cell death has revealed that the virus triggers programmed cell death through interactions between specific viral components and host cells, with different viral proteins and nucleic acid species selectively activating distinct cell death pathways. IAV-induced apoptosis is mediated primarily through viral protein interactions with mitochondrial and nuclear regulatory pathways. The viral protein PB1-F2 directly interacts with the mitochondrial proteins ANT3 and VDAC1, disrupting mitochondrial membrane permeability and triggering the intrinsic (mitochondrial) apoptotic pathway, thereby enhancing viral pathogenicity [7]. Similarly, the nucleoprotein (NP) promotes apoptosis through two complementary mechanisms: first, by targeting the E3 ubiquitin ligase RNF43 to regulate p53 ubiquitination, leading to p53 stabilization and transcriptional activation of pro-apoptotic genes [8]; and second, by interacting with the chaperone protein clusterin, thereby weakening its inhibitory binding to the pro-apoptotic protein BAX, which facilitates BAX translocation to mitochondria and initiates the apoptotic cascade [9]. In contrast, virus-derived nucleic acid–mediated necroptosis represents a distinct pathway. In addition to viral proteins, Z-RNA—an aberrant RNA structure generated during IAV replication—can activate the nucleic acid sensor ZBP1 (Z-DNA-binding protein 1) within infected cells. Upon activation, ZBP1 triggers necroptosis through the sequential activation of the ZBP1–RIPK3–MLKL signaling axis, ultimately leading to regulated necrotic cell death [10]. Interestingly, with the recent introduction of the concept of PANoptosis, ZBP1 has been shown to not only play a role in necroptosis but also participate in regulating apoptosis and pyroptosis signaling pathways. PANoptosis is a unique inflammatory form of programmed cell death characterized by the simultaneous activation of multiple death pathways. Unlike individual cell death pathways, PANoptosis provides a comprehensive immune response during pathogen infection [11]. As early as 2016, Kuriakose et al. discovered that ZBP1 can serve as a specific sensor for IAV, regulating the activation of NLRP3 inflammasomes and PANoptosis through the RIPK1-RIPK3-Caspase-8 axis [12]. This leads to an inflammatory cytokine storm in the later stages of the disease, causing damage to tissues and organs.

The novel coronavirus (SARS-CoV-2), which caused a global pandemic in 2019, also triggers multiple forms of cell death and worsens the patient’s condition through a cytokine storm, although its specific mechanisms differ from those of IAV. Regarding apoptosis, SARS-CoV-2 may evade the host’s clearance mechanisms by inhibiting apoptosis, allowing early viral replication and spread [13]. Necroptosis and its connection to SARS-CoV-2 infection have also garnered significant attention. Xu et al. studied the role of RIPK1 in viral infection and found that NSP12, a key component of the SARS-CoV-2 replication and transcription machinery, not only directly participates in viral RNA replication but also promotes RIPK1 activation, which drives inflammation and increases the expression of host viral receptors such as ACE2 and EGFR [14]. This ultimately enhances viral replication and spread. Inflammasomes play a crucial role in the cytokine storm triggered during the later stages of SARS-CoV-2 infection. Several proteins encoded by SARS-CoV-2 regulate inflammasome activity. Interestingly, experiments have shown that, in addition to GSDM family proteins and MLKL—which impair cell integrity by forming membrane pores—the E glycoprotein encoded by SARS-CoV-2 can also form cation channels, leading to rapid cell death [15]. Whether it is IAV or SARS-CoV-2, ongoing research continues to deepen our understanding of these viruses. Notably, recent studies indicate that in critically ill patients infected with either virus, there is a significant increase in the expression of the membrane rupture mediator protein NINJ1. Both viruses involve PANoptosis, and any of the death pathways can independently activate the NINJ1 protein [16], further intensifying the inflammatory response.

In addition to the two most extensively studied pathogens mentioned above, cell death caused by other pathogens also significantly harms the body. For example, Middle East Respiratory Syndrome Coronavirus (MERS-CoV) can activate both endogenous and exogenous apoptosis pathways simultaneously, leading to typical pathological features of severe lung injury [17]. Human Immunodeficiency Virus type 1 (HIV-1), a major global health challenge, has a pathogenic mechanism that specifically targets CD4^+^ T lymphocytes, ultimately resulting in Acquired Immunodeficiency Syndrome (AIDS). In programmed cell death, apoptosis pathways serve dual biological roles: on one side, they function as a host antiviral defense to prevent viral spread, and on the other side, they contribute to CD4^+^ T cell depletion [18]. Studies indicate that HIV-1 can induce apoptosis through multiple pathways: its viral proteins Vpr, Tat, and Nef can directly activate apoptosis signaling pathways, while also indirectly causing immune cell death via bystander effects and a systemic inflammatory environment. This apoptosis induction mechanism, involving both direct and indirect effects, forms the core molecular basis of HIV-1-related immune damage, pushing the disease toward its advanced stages [19]. In Ebola virus infection, although apoptosis and necrosis are recognized as the main modes of cell death, additional research shows that Ebola virus can also initiate pyroptosis and necroptosis in infected cells through direct contact. Furthermore, the virus can release soluble mediators that indirectly induce pyroptosis or necroptosis in uninfected bystander cells [20].

In addition to viruses, many common bacteria are also closely linked to host cell death mechanisms. For example, Staphylococcus aureus can evade cell death processes that eliminate pathogens by producing virulence factors and other adaptive strategies. When it needs to propagate and spread, it triggers pyroptosis, necroptosis, or necrosis by lysing host cells to release progeny bacteria, which then infect new targets [21]. Mycobacterium tuberculosis blocks various forms of programmed cell death by interfering with death receptor pathways or controlling key apoptotic proteins, creating a stable environment for replication within the host cell [22]. Yersinia species deploy multiple effector proteins (YopJ, YopM, YopK) as “brakes” to suppress the host’s defense cell death pathways, supporting the ongoing infection [23]. These examples collectively demonstrate the close relationship between pathogens and cell death mechanisms. By precisely activating or inhibiting these pathways to adapt to the host environment, pathogens establish colonization and facilitate spread within the host, ultimately causing severe tissue damage and clinical symptoms.

The interplay between viruses and cell death is highly complex. During the viral replication phase, to ensure efficient propagation, viruses often actively suppress host cell death by encoding multiple effector proteins that target and block key nodes within cell death pathways. For example, cytomegalovirus encodes a series of intrinsic inhibitory factors that sequentially antagonize necroptosis, extrinsic apoptosis, and intrinsic apoptosis in infected cells [24]. Therefore, whether viruses inhibit or induce cell death depends on the viral type, pathogenic characteristics, stage of infection, and the specific cellular microenvironment, and cannot be generalized [25].

### 2.2. Cell Death Mechanisms Involved in PMR

Pathogen-induced lytic cell death is a current research hotspot, representing a critical link between innate immunity and pathological injury. PMR is a key terminal event in this process. Its conceptual origins can be traced back to the late nineteenth century, when Rudolf Virchow introduced the concept of necrosis to describe tissue death [26]. Microscopic observations revealed that necrotic cells typically exhibit swelling, plasma membrane rupture, and leakage of intracellular contents. Following PMR, osmotic imbalance between the intracellular and extracellular environments leads to irreversible changes in both the nucleus and cytoplasm [27]; thus, PMR is widely regarded as a contributing factor to disease pathogenesis.

Pyroptosis is a form of inflammasome-triggered programmed cell death characterized by the loss of plasma membrane integrity. GSDMD is cleaved by caspase-1 as well as caspase-4/5/11, and its N-terminal fragment rapidly targets membrane components of macrophages, inducing pore formation in the plasma membrane [28,29]. However, a study by Kayagaki et al. in 2021 demonstrated that GSDMD pores are insufficient in size to permit the release of large molecules such as LDH, suggesting that PMR may instead depend on the activity of the protein NINJ1 [3]. Necroptosis is another form of regulated necrosis that, similar to pyroptosis, is tightly controlled by specific genetic and molecular pathways, and whose key executioner proteins can oligomerize to form membrane pores. Its activation requires the involvement of RIPK3 and MLKL and can be induced by multiple mediators, including death receptors, interferons, and Toll-like receptors, making it one of the most extensively studied forms of regulated necrosis [30]. In addition to pyroptosis and necroptosis, ferroptosis also leads to loss of membrane integrity and is characterized by the iron-dependent accumulation of toxic lipid reactive oxygen species (ROS) [31] and the manifestation of necrosis-like morphological features [32].

Pyroptosis, necroptosis, and ferroptosis represent the most extensively studied forms of lytic cell death underlying PMR. Other types of programmed cell death, such as parthanatos, cuproptosis, and NETosis, can also result in membrane disruption but are not discussed in detail in this review.

### 2.3. Formatting of Mathematical Components

Under physiological conditions, PMR can be observed in processes such as muscle contraction, cell migration, division, and fusion [33], and is often quickly repaired by complex internal cellular mechanisms, transforming it into a vital force driving development, movement, and tissue repair. However, during infection or inflammation, plasma membrane damage caused by pathogens or immune cells can negatively impact cell fate [34]. For example, pathogen-driven lytic cell death can trigger a strong inflammatory response through plasma membrane rupture in the final stages. The Damage-Associated Molecular Patterns (DAMPs) released after plasma membrane rupture can be recognized by pattern recognition receptors (PRRs) on neighboring cells, thereby activating inflammatory signaling pathways and promoting the release of chemokines and cytokines. These mediators can attract more inflammatory cells and initiate adaptive immune responses, creating a self-amplifying loop of inflammation [35,36]. Inflammation is essentially the body’s adaptive response to harmful stimuli, aiding in pathogen clearance and initiating repair; however, excessive or chronic inflammation can disturb homeostasis, contributing to the development and progression of various diseases, including autoimmune diseases, cardiovascular diseases, cancers, and neurodegenerative diseases [37]. Studies have shown that in patients infected with the novel coronavirus, plasma levels of molecules regulating monocyte activation and migration, as well as γ-interferon (IFN-γ) levels, are significantly elevated compared to healthy controls, indicating the presence of abnormally activated inflammatory responses, possibly related to viral-induced membrane damage and the release of associated DAMPs [38]. Besides inflammatory pathways, PMR can also directly cause tissue damage through other mechanisms. For instance, in the liver, high levels of bile acids can directly harm membrane lipid structures and induce oxidative stress, damaging hepatocyte plasma membranes. This process is a key event in the development of cholestatic liver disease [39]. Overall, plasma membrane rupture acts like a double-edged sword: in the early stages, it helps initiate immune clearance, but in later stages, it can lead to tissue damage due to excessive amplification of inflammatory signals. The specific outcome depends on the extent of damage, the cell’s ability to repair, and the regulatory signals present in the microenvironment.

## 3. Passive Regulatory Mechanisms of PMR

The plasma membrane plays a crucial role in maintaining the homeostasis of mammalian cells. It not only acts as a physical barrier that separates the intracellular and extracellular environment, preserving the cell’s structural integrity, but it also mediates extracellular signal transduction, immune recognition, ion and small molecule transport, and participates in vital physiological processes such as nutrient uptake and vesicular transport (e.g., endocytosis and exocytosis) [40]. Therefore, the loss of plasma membrane integrity will lead to the termination of cell life and may facilitate the spread of pathogens within the host. Traditionally, plasma membrane rupture is viewed as a passive lytic process caused by osmotic imbalance. A classic biological example is hemolysis of red blood cells in hypotonic solutions: under isotonic conditions, red blood cells can normally perform oxygen transport; however, when environmental osmotic pressure drops below a certain level, a large influx of water causes membrane rupture, hemoglobin release, and subsequent hemolytic diseases. Similarly, passive plasma membrane rupture mainly occurs through two pathways: one is triggered by the activation of internal cell death programs, such as pyroptosis and necroptosis; the other results from external damaging factors, including mechanical injury, radiation, or intense and prolonged disturbances in the microenvironment [40,41]. The common final step in these processes is the loss of membrane integrity and the disruption of osmotic balance. The following discussion will systematically explore the specific molecular mechanisms underlying plasma membrane rupture, starting with key regulatory molecules and signaling pathways, and discuss their role in infection and disease progression.

### 3.1. MLKL

MLKL is a key executioner of plasma membrane permeabilization during necroptosis [27]. Structurally, it comprises an N-terminal four-helix bundle (4HB) domain, a C-terminal pseudokinase (PsK) domain, and a brace region of two helices that links these domains and transduces phosphorylation signals from the PsK to the 4HB, thereby enabling cytotoxic activation and coordinating conformational changes [27,42,43]. RIPK3-mediated phosphorylation induces MLKL oligomerization and membrane translocation, ultimately leading to membrane disruption. Although caspases are not directly involved in necroptosis, caspase-8 inhibits this pathway by cleaving RIPK1 or forming heterodimers with c-FLIP, thereby blocking signal transduction; accordingly, inhibition of caspase-8 permits necroptosis to be activated as an alternative defense mechanism [44,45,46]. Under these conditions, RHIM domain-containing proteins (e.g., RIPK1, ZBP1, and TRIF) promote necrosome assembly, driving RIPK3 oligomerization and autophosphorylation, which in turn phosphorylates the PsK domain of MLKL to trigger its membrane-disrupting activity [47,48].

Activated MLKL undergoes oligomerization and targets membranes via electrostatic interactions between positively charged residues within the 4HB domain and phospholipids [49]. In addition to the plasma membrane, phosphorylated MLKL can assemble on lysosomal membranes, promoting lysosomal clustering, fusion, and permeabilization, indicating a broader membrane-targeting capacity [50]. Mechanistically, several models have been proposed for MLKL-mediated membrane disruption: direct formation of hydrophilic pores permitting passage of macromolecules (~10 kDa) and causing osmotic lysis [51]; assembly into amyloid-like fibers on membranes, leading to mechanical damage or recruitment of effector molecules [52]; or formation of ion-selective channels that disturb ionic homeostasis. Overall, MLKL oligomerization compromises membrane integrity, resulting in osmotic imbalance, cell swelling, and eventual rupture.

### 3.2. GSDMs

The Gasdermin (GSDM) family comprises conserved mammalian pore-forming proteins that execute membrane permeabilization during lytic cell death processes such as pyroptosis. This family was first identified in mice by Saeki et al. in 2000 [53]. During evolution, gene duplication and loss events have resulted in species-specific repertoires of GSDM members (six in humans: GSDMA–GSDME and PJVK; approximately ten in mice) [54]. Except for PJVK, all GSDMs share a conserved architecture consisting of an N-terminal (NT) pore-forming domain and a C-terminal (CT) autoinhibitory domain, connected by a protease-sensitive linker [54,55]. Proteolytic cleavage relieves autoinhibition, releasing the NT domain, which oligomerizes and forms pores in the plasma membrane, thereby disrupting ionic homeostasis and driving pyroptosis. These processes play important roles in infection, autoinflammatory disorders, and cancer [55,56]. Beyond membrane rupture, GSDMs also possess immunomodulatory functions; for example, GSDMD-NT promotes the release of neutrophil extracellular traps (NETs) [57]. Most GSDM family proteins exist in an autoinhibited state, in which the CT domain suppresses NT activity, reflecting a shared molecular regulatory mechanism within the family [56].

During pathogen infection, GSDMD serves as the principal effector of pyroptosis. In the canonical pathway, inflammasomes activate caspase-1 via ASC-dependent or CARD-containing complexes. Activated caspase-1 cleaves GSDMD at Asp275, generating an approximately 30 kDa NT fragment, while concurrently processing pro-inflammatory cytokines such as IL-1β and IL-18 [56,58,59]. In the noncanonical pathway, lipopolysaccharide (LPS) directly activates caspase-4/5 (in humans) or caspase-11 (in mice) to cleave GSDMD [59]. Additionally, under certain conditions, caspase-8 can directly cleave GSDMD, integrating it into broader cell death signaling networks [54,60]. The released NT fragment binds to the phospholipid bilayer and oligomerizes to form membrane pores [29], with a preference for acidic phospholipids [61]. These pores permit the release of cytokines such as IL-1β [62,63]. Notably, pore formation alone is often insufficient to induce complete cell lysis; instead, GSDMD-mediated ionic and osmotic imbalance can activate and recruit another executor of plasma membrane rupture, NINJ1. NINJ1 then drives extensive membrane rupture, lactate dehydrogenase (LDH) release, and DAMP efflux, thereby amplifying inflammatory responses and linking pore formation to terminal membrane disintegration [3].

### 3.3. Lipid Peroxidation of the Plasma Membrane Phospholipid Bilayer

Peroxidation of phospholipids in the plasma membrane bilayer represents a key mechanism regulating membrane integrity and cell fate [31]. Under oxidative stress, phospholipids are oxidized into lipid hydroperoxides and reactive aldehydes; moderate oxidation can support signal transduction, whereas excessive damage disrupts membrane structure, leading to plasma membrane rupture (PMR) and cell death. Ferroptosis is the most extensively studied form of such cell death, directly triggered by iron-dependent lipid peroxidation [64]. Although the term was formally defined in 2012, earlier concepts such as “oxytosis,” as well as studies on carbon tetrachloride toxicity, had already implicated lipid peroxidation as a major driver of cell death [65,66].

Lipid peroxidation proceeds via enzymatic, free radical, and non-radical pathways [67]. Under physiological conditions, products such as lipid hydroperoxides are efficiently cleared by antioxidant systems, including glutathione peroxidase 4 (GPX4). However, inhibition of GPX4 activity or depletion of antioxidant reserves disrupts redox homeostasis and triggers ferroptosis. Sustained lipid peroxidation compromises membrane fluidity, disrupts ionic homeostasis, and induces organelle dysfunction, ultimately leading to PMR and contributing to pathological processes such as neurodegenerative diseases, cancer, and ischemia–reperfusion injury [31]. Beyond ferroptosis, lipid peroxidation can also promote PMR in other contexts. For example, in mitochondrial permeability transition (MPT)-mediated necrosis, oxidative stress serves as a key trigger. Mitochondrial permeability transition refers to the formation of non-selective channels in the inner mitochondrial membrane under conditions such as Ca^2+^ overload or oxidative stress, allowing the passage of small molecules [68,69]. Under Ca^2+^ overload or oxidative stress [70], the mitochondrial permeability transition pore (mPTP) remains persistently open in a high-conductance state, leading to NADH depletion and consequent dysfunction of the respiratory chain [71]. Concurrently, the imbalanced distribution of ions and molecules causes mitochondrial matrix swelling [68], while the permeability of the outer mitochondrial membrane (OMM) is altered under the regulation of BAX/BAK [72], ultimately culminating in plasma membrane rupture.

## 4. Active Regulation of PMR by NINJ1

For a long time, the exact mechanism of plasma membrane rupture (PMR) at the final stage of lytic cell death has been a major topic of debate, with traditional views mostly seeing it as a passive event caused by osmotic imbalance. However, a groundbreaking study published in Nature in 2021 revealed for the first time that the plasma membrane protein NINJ1 undergoes oligomerization during cell death and actively causes the disruption of plasma membrane integrity [3]. This discovery fundamentally changed previous ideas and started a new chapter in understanding actively regulated mechanisms behind PMR.

### 4.1. Structure and Function of NINJ1

NINJ1 (nerve injury–induced protein 1) was first identified in 1996 and was found to be upregulated in neurons and Schwann cells following axonal injury, where it participates in peripheral nerve repair by mediating homophilic adhesion [1]. NINJ1 is a member of this group and has a molecular weight of 16.3 kDa, exhibiting a typical transmembrane topology in which both the N- and C-termini are located extracellularly, with two transmembrane domains (at amino acid residues 72–100 and 118–139, respectively) and an intervening intracellular segment [3,73,74]. Subsequent studies have shown that NINJ1 mediates intercellular adhesion through the Pro26–Asn37 region within its N-terminal extracellular domain, a function that depends on the specific recognition capability of a conserved tryptophan–arginine cluster motif within this region. Further mechanistic investigations confirmed that, through this specific homophilic adhesion, NINJ1 regulates interactions between Schwann cells and neurons during peripheral nerve regeneration, thereby promoting nerve repair [1,74].

**Figure 1:**
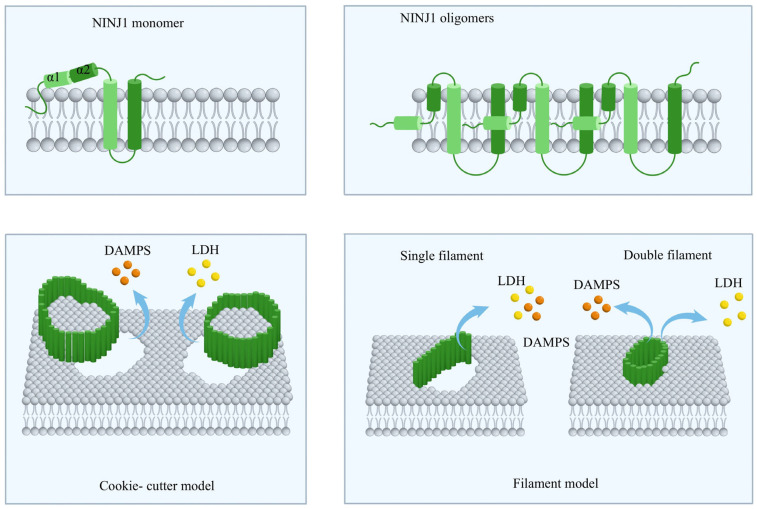
The structure of NINJ1 monomers and oligomers, and two models of the membrane rupture mechanism. Cookie-cutter model: Plasma membrane rupture via hole-punching in the cell membrane; Filament model: Membrane rupture through unilateral or bilateral fibril-mediated incision of the cell membrane. This ultimately leads to the release of DAMPs, LDH, and other substances.

Beyond its role in adhesion, NINJ1’s active participation in plasma membrane rupture has garnered increasing interest recently. Notably, although NINJ1 belongs to the same class of membrane-disrupting proteins as pore-forming proteins like MLKL and GSDMs, its function does not involve forming localized membrane pores but instead triggers a large-scale loss of plasma membrane integrity, ultimately causing complete cell lysis. A cryo-electron microscopy study published in 2023 revealed the high-resolution structure of NINJ1 in its resting state, showing it exists as a soluble monomer anchored to the plasma membrane, with an extracellular region featuring characteristic kinked α1–α2 helices and a transmembrane region made up of α3–α4 helices forming a hairpin embedded in the lipid bilayer [73,75]. Upon receiving activation signals, NINJ1 undergoes a process called oligomerization through a cis-promoting interaction between its intracellular domain and N-linked glycosylation at Asn60, assembling into homomeric complexes composed of two to six NINJ1 monomers [76]. Over time, these complexes further polymerize into higher-order structures, creating amphipathic filamentous polymers on the cell membrane that facilitate NINJ1-mediated membrane rupture (Figure 1) [73]. Notably, mutations in key residues such as Lys45 significantly impair filament formation and cytotoxicity, confirming that oligomerization forms the structural basis of NINJ1-mediated membrane rupture [3,73], while also offering new therapeutic perspectives for related diseases.

In addition to promoting nerve repair and mediating plasma membrane rupture, the autoinhibitory function of NINJ1 is also very important because it helps explain how intracellular NINJ1 keeps its shape under normal conditions and allows for hypotheses about how it becomes activated. Among pore-forming proteins, the autoinhibitory mechanism of GSDMD limits its natural ability to cause pyroptosis, thus preventing pyroptotic cell death [77]. Similarly, NINJ1 forms face-to-face homodimers with an unbent TM1 three-helix fold, where autoinhibition is maintained by a central polar pocket made up of residues S46, E49, D116, N119, N120, and Y41, K44, Q45. Trans-autoinhibition occurs through mutual shielding of hydrophilic surfaces. When specific signals appear, this dimerization breaks apart, ultimately switching NINJ1 into a membrane-disrupting mode [78].

Beyond its direct role in membrane rupture, NINJ1 also has an important function in neurological disorders. Studies have shown that NINJ1 knockout mice display obsessive–compulsive–like behaviors along with reduced glutamate levels in the brain, indicating a role for NINJ1 in regulating neurotransmitter balance and a possible connection to the mechanisms behind neuropsychiatric disorders [79].

### 4.2. Molecular Mechanisms of NINJ1-Mediated PMR

Although the central role of NINJ1 in mediating plasma membrane rupture has been established, the exact molecular mechanisms by which its oligomerization causes loss of membrane integrity, controls the release of intracellular contents, and influences inflammatory responses have remained only partially understood for a long time [75]. Recent models consistently suggest that NINJ1 consists of amphipathic extracellular helices (α1 and α2) and transmembrane helices (α3 and α4), with the insertion of α1 and α2 changing membrane surface properties, while conserved glycine residues within α3 and α4 (G93, G95, and G124) provide the structural flexibility needed for conformational changes [73,80].

Currently, several structural models supported by experimental data and imaging evidence have been proposed to explain how NINJ1 disrupts the plasma membrane. Degen and colleagues introduced a “filament” model, suggesting that NINJ1 operates through two mechanisms: first, a single hydrophilic filament directly alters lipid order and creates perforations in the membrane; second, two antiparallel filaments stack to form a zipper-like structure, where the hydrophobic interface interacts with the membrane while the hydrophilic interior acts as a conduit, ultimately causing content release via an “unzipping” mechanism (Figure 1) [73,77]. In 2024, the David group proposed a “cookie-cutter” model based on live-cell super-resolution imaging and in vitro reconstitution experiments, showing that NINJ1 oligomers assemble into ring-like structures on the membrane that cut out and release membrane patches in a mold-like fashion, leading to significant release of LDH, DAMPs, and HMGB1 (Figure 1) [80]. Follow-up cryo-electron microscopy studies supported this model by demonstrating that NINJ1 can form cyclizable linear filaments that rupture membranes by wrapping and solubilizing membrane fragments. Conversely, the homologous protein NINJ2 does not effectively cyclize because its filaments bend toward the cytosolic side, resulting in the loss of PMR-mediating capacity and further emphasizing the unique structure of NINJ1 [81]. Notably, a recent study by Hartenian et al. further supports the mechanism by which NINJ1 double-stranded filaments open the plasma membrane in a zipper-like manner through molecular dynamics simulations. In this study, two coarse-grained molecular dynamics systems were constructed for comparison. In simulations of the “cookie-cutter” model, NINJ1 filaments encircled lipid nanodiscs and were forced to bend toward the hydrophobic face; this geometric configuration disrupted lateral interactions between adjacent subunits, resulting in multiple discontinuities within the filament and ultimately leading to structural disassembly. In contrast, in simulations of the “zipper” pore model, NINJ1 filaments bent toward the hydrophilic face, thereby maintaining structural integrity and stability [82].

Based on the available evidence, we find that the mechanism of action of NINJ1 shares certain similarities with the formation of “toroidal lipid pores.” Such pores are not characterized by rigid protein walls but instead develop from protein–lipid interactions that induce local membrane curvature and lipid reorganization [83]. Consistent with this idea, recent biomechanical studies have shown that oligomerized NINJ1 significantly decreases the mechanical stability of the plasma membrane, making it more susceptible to rupture under lower levels of stress [84]. Based on these findings, we proposed a hypothesis that NINJ1 promotes localized lipid reorganization within the plasma membrane to create unstable membrane microdomains resembling toroidal lipid pores, thereby changing membrane physical properties and ultimately causing catastrophic rupture under osmotic or mechanical stress. This hypothesis offers a new perspective for understanding the membrane-disruptive mechanism of NINJ1, although its molecular details still need to be experimentally confirmed.

### 4.3. PMR Mechanisms of NINJ1 Activation

Upon activation, NINJ1 quickly undergoes oligomerization and then disrupts plasma membrane integrity, often causing a strong inflammatory response. Therefore, understanding the mechanisms behind NINJ1 activation is highly important. Several pathological events caused by pathogen infection, such as homeostatic imbalance and abnormal molecular signaling pathways, may involve key steps that lead to NINJ1 activation. Clarifying how this protein is regulated will provide a crucial theoretical foundation for preventing and treating related diseases. However, the mechanisms proposed for NINJ1 activation are varied and, in some cases, even more complex than those explaining its membrane-disrupting activity.

The relationship between ion flux and NINJ1 activation appears to be indirect rather than a direct causal trigger. In ferroptosis, for example, Ca^2+^ influx occurs upstream of NINJ1 oligomerization and plasma membrane rupture (PMR); interventions such as Ca^2+^ chelators or calcium channel blockers effectively reduce PMR events, indicating that Ca^2+^ signaling functions as an upstream trigger [85]. Mechanistic studies further show that NINJ1 is not directly activated by Ca^2+^ influx but instead acts downstream of it: NINJ1 is dispensable for early ferroptotic events—including lipid peroxidation, channel-mediated Ca^2+^ influx, and cell swelling—yet is essential for the subsequent loss of plasma membrane integrity [85]. In addition, Ca^2+^ overload can induce mitochondrial stress, which in turn activates the p53/NINJ1 pathway, ultimately leading to plasma membrane rupture in acinar cells [86]. Borges et al. further demonstrated that extracellular ATP can activate NINJ1 by inducing Ca^2+^ influx and TMEM16F-dependent lipid scrambling, thereby causing cell lysis independently of inflammasomes, pannexin-1, and GSDMD [87]. Collectively, these findings indicate that ion flux does not directly activate NINJ1 but instead regulates it indirectly through intermediate processes such as mitochondrial stress and lipid perturbation.

Overall, current hypotheses about how NINJ1 activation leads to plasma membrane rupture can be broadly categorized into two main models. First, NINJ1-mediated membrane rupture is thought to be regulated by signaling pathways, although the specific proteins involved have not yet been clearly identified. Second, this process may not be driven by signaling cascades but rather by mechanical forces that ultimately weaken membrane integrity. Regardless of the mechanism, pathogen infection is a critical factor that must not be overlooked. Hypotheses regarding the signaling pathways regulating NINJ1 activation suggest that caspase-8 may promote NINJ1 oligomerization through specific signaling cascades during inflammatory cell death, thereby providing an alternative activation route independent of ion flux [88]. At the level of transcriptional regulation, activation of NF-κB upregulates NINJ1 expression in human endothelial cells (HECs): tumor necrosis factor TNF-α binds to TNFR1 and indirectly activates the NF-κB signaling pathway, leading to increased transcription and translation of NINJ1 [89]. Similarly, as an example of transcriptional control, NINJ1 is a target gene of p53 and participates in a p53–NINJ1 feedback loop that regulates p53 mRNA translation as well as p53-dependent premature senescence, cell proliferation, apoptosis, and radiation-induced cell death [6,90]. In addition, NINJ1 is involved in multiple other signaling processes. In pregnancy-associated trophoblast cells, elevated NINJ1 expression suppresses the STAT3 and Notch signaling pathways, contributing to adverse pregnancy outcomes [91,92]. In contrast, in human lung adenocarcinoma (LUAD) tissues and cell lines, increased NINJ1 expression exerts pro-tumorigenic effects by directly binding to PI3K and activating downstream AKT signaling [93].

However, a recent study by Xu et al. found that treating bone marrow–derived macrophages (BMDMs) with the caspase-8 inhibitor Z-IETD-FMK alone could not fully suppress NINJ1 oligomerization or lactate dehydrogenase (LDH) release, whereas the pan-caspase inhibitor Z-VAD-FMK more effectively inhibited NINJ1 oligomerization [16]. This suggests that activation of any single PANoptosis pathway—pyroptosis, apoptosis, or necroptosis—is enough to trigger NINJ1 oligomerization. Only blocking all three pathways at once can completely prevent NINJ1 oligomerization and subsequent cell lysis. Meanwhile, a 2025 serological study in COVID-19 patients provided indirect evidence linking NINJ1 activation to cytokine-driven bystander inflammation: serum NINJ1 levels in 264 patients showed strong positive correlations with inflammatory cytokines (IL-6, TNF-α, IFN-γ) and inflammatory markers (CRP, PCT, ferritin, LDH), and these correlations increased with disease severity [94]. Taken together, these findings indicate that NINJ1 activation is regulated by multiple signaling pathways and cell death programs, and it may represent a convergent therapeutic intervention node in pathogen infection; however, this concept still requires further functional validation.

However, other reports favor a model in which intrinsic physical changes in the membrane itself drive NINJ1 activation and PMR, with mechanisms described in greater detail than the aforementioned models. In a 2023 study by Dondelinger et al., wild-type and NINJ1-deficient mouse embryonic fibroblasts (MEFs) were subjected to hypotonic shock to demonstrate that cell swelling alone is sufficient to trigger the “functionalization” or “opening” of NINJ1 oligomers [95]. Notably, more recent work by Hartenian et al. has clearly distinguished NINJ1 “oligomeric assembly” from “functional pore opening” as two independent steps. They showed that inhibition of cell swelling completely blocks PMR, while NINJ1 oligomerization remains largely unaffected. Consistent evidence from atomic force microscopy measurements and molecular dynamics simulations indicates that membrane tension generated during swelling is the key physical factor driving the transition of pre-assembled NINJ1 double-stranded filamentous oligomers from an inactive, closed conformation to an active, open state that mediates PMR, whereas swelling alone is insufficient to induce NINJ1 oligomerization. Furthermore, studies on the autoinhibitory mechanism of NINJ1 have revealed that the dissociation of face-to-face NINJ1 dimers may be induced by plasma membrane structural changes associated with cell death [82], suggesting that the mechanical properties of the membrane (e.g., curvature alterations) may also contribute to NINJ1 activation. In addition, mechanical force itself has recently been independently supported as a direct activator of NINJ1: using a reproducible strain-based 384-well cell stretching system, the Zhu group performed a small interfering RNA (siRNA) screen targeting transmembrane proteins and identified NINJ1 as a key regulator of plasma membrane fragility under mechanical stress [84]. Despite these advances, the precise cascade by which NINJ1 transitions from an autoinhibited state to full activation and executes membrane disruption remains incompletely understood.

Based on the available evidence, we propose a comprehensive mechanism to explain the activation of NINJ1 and its pathological roles during pathogen infection. Pathogen invasion, through recognition of PAMPs and DAMPs, activates multiple cell death signaling pathways (PANoptosis) and induces ionic imbalance (such as Ca^2+^ overload), while simultaneously causing cell swelling and changes in the physical properties of the plasma membrane. These signals collectively relieve the autoinhibitory state of NINJ1 and trigger its oligomerization. Oligomerized NINJ1 not only directly damages lipid membrane integrity but also significantly decreases the membrane’s mechanical stability, making it more prone to rupture under osmotic pressure or mechanical stress from tissues. After plasma membrane rupture, the large release of DAMPs further amplifies local and systemic inflammation by recruiting and activating additional immune cells, creating a positive feedback loop of “cell death–NINJ1 activation–DAMP release–inflammatory amplification” that ultimately leads to tissue damage and disease progression. This model also clarifies why NINJ1-mediated damage is especially severe in mechanically active organs such as the lung and heart [84].

## 5. Development of NINJ1-Targeted Therapeutic Strategies to Inhibit PMR

NINJ1 plays a vital role in the development of various diseases, including inflammation and cancer, by mediating lytic cell death pathways. NINJ1-induced plasma membrane rupture results in the large-scale release of inflammatory cytokines and damage-associated molecular patterns (DAMPs) [3], which provoke systemic inflammatory responses and worsen disease progression. Severe acute pancreatitis (SAP), for instance, is one of the most common gastrointestinal reasons for hospitalization, and abnormal intracellular calcium (Ca^2+^) signaling in acinar cells is a key mechanism in its development [96]. Studies by Lee et al. showed that NINJ1 expression is controlled by intracellular Ca^2+^ levels in acinar cells, and removing NINJ1 genetically delays plasma membrane rupture and reduces the severity of SAP [86]. A recent study further clarified NINJ1’s role during influenza A virus (IAV) infection, revealing that IAV infection significantly increases NINJ1 expression in macrophages, and that blocking NINJ1 effectively prevents plasma membrane rupture, ultimately reducing IAV-associated lung injury and death without impacting the course of cell death or early inflammatory responses.

In severe infectious diseases like sepsis and COVID-19, systemic coagulation often occurs alongside inflammatory reactions [97]. Studies have demonstrated that blocking NINJ1 significantly decreases platelet-dependent thrombosis and the development of disseminated intravascular coagulation (DIC) in sepsis models [88].

Beyond inflammatory diseases, NINJ1 also plays a significant role in tumor initiation and progression. A close regulatory link exists between NINJ1 and the tumor suppressor p53, whereby the loss of NINJ1 suppresses cell proliferation by promoting p53 mRNA translation, while also enhancing apoptosis and premature senescence [6]. Notably, the role of NINJ1 in cancer seems to depend on the genetic status of p53. In p53-mutant cells, NINJ1 deficiency further raises mutant p53 protein levels, which boosts cell growth and migration, whereas in p53 wild-type cells, NINJ1 loss inhibits these malignant traits [98].

As a protein originally identified in the central nervous system, NINJ1 is also involved in the development of various neurological disorders. Besides the obsessive–compulsive–like behaviors mentioned earlier, research has shown that NINJ1 expression increases in experimental autoimmune encephalomyelitis (EAE) models, indicating a possible role in the disease’s progression [99].

Taken together, NINJ1 plays crucial roles in various disease processes, and developing targeted therapies against NINJ1-mediated plasma membrane rupture has become a major focus of current research. The following sections will systematically review the therapeutic strategies targeting NINJ1 and the latest progress in this field.

### 5.1. Monoclonal Antibody

After discovering the role of NINJ1 protein in mediating plasma membrane rupture, Kayagaki’s research team successfully developed a monoclonal antibody that specifically targets the mouse NINJ1 protein. This antibody effectively blocks the PMR process by inhibiting NINJ1 oligomerization. Among them, clone D1 and clone 25 are two representative monoclonal antibodies: clone D1 specifically recognizes the C-terminal 141–152 amino acid residues of the extracellular region of mouse NINJ1, while clone 25 targets the N-terminal 22–31 residues. Notably, neither of them binds to the human NINJ1 protein, demonstrating strict species specificity. Mechanistic studies show that these antibodies inhibit the formation of higher-order oligomers of NINJ1 through steric hindrance, thereby preventing their assembly into filamentous structures with cytolytic activity. Importantly, this inhibitory effect does not interfere with the progression of cell death itself but specifically intervenes in the plasma membrane rupture process after cell death [100]. This work theoretically demonstrates the feasibility of targeting specific epitopes within the extracellular domain of NINJ1 to prevent oligomerization and thereby block PMR, and provides valuable antibody tools for mechanistic studies and target validation in experimental models. It should be noted that the species specificity and in vivo pharmacokinetic properties of existing antibodies have not yet been fully optimized; thus, they are currently more suitable for preclinical mechanistic investigations, and significant challenges remain before clinical translation can be achieved.

### 5.2. Small Molecule Inhibitors

#### 5.2.1. Inhibition of NINJ1 Oligomerization

The cytoprotective effect of glycine is an evolutionarily conserved biological phenomenon widely observed in various cell types. Since this effect was first reported in the last century, numerous studies have demonstrated that glycine significantly increases the resistance of various cells to necrotic cell death and pyroptosis [101]. Early research even proposed that glycine might exert its protective effects by preventing the formation of hydrophilic membrane pores [102]. In the context of NINJ1-mediated lytic cell death, Borges and colleagues utilized this property of glycine and confirmed that it could effectively inhibit NINJ1 oligomerization on the plasma membrane. This discovery not only uncovers a new mechanism for glycine’s cytoprotective effects but also offers an important theoretical foundation for designing small-molecule inhibitors that target the NINJ1 aggregation process [103]. Mechanistic studies suggest that glycine directly interacts with NINJ1, blocking the formation of its oligomers while preserving the secondary structure stability of the N-terminal α-helix and its inherent membrane-lytic function. This unique mechanism enables glycine to maintain specific cellular functions while safeguarding the structural integrity of the plasma membrane [103]. However, the precise molecular details of how glycine interacts with NINJ1 remain unclear, and further research is necessary to identify its binding site, mode of action, and regulatory network.

A related compound is muscimol, a classic agonist of neuronal GABAA receptors. Muscimol exhibits a mechanism similar to that of glycine in inhibiting NINJ1: both compounds specifically prevent the oligomerization of NINJ1 in pyroptotic cells and effectively block pyroptotic plasma membrane rupture without affecting pore formation mediated by gasdermin D or the release of inflammatory factors like IL-1β [103,104]. Notably, the protective effects of these two compounds are reversible—although the treated pyroptotic cells maintain plasma membrane integrity, they enter a metabolically quiescent state and are essentially in biological death. However, due to the high concentrations needed for effectiveness and their multi-target effects, these molecules may not have clinical translational potential [104]. These findings are currently limited to validating the feasibility of small-molecule interventions and have not yet progressed to therapeutic development; however, they provide a theoretical basis for considering NINJ1 oligomerization as a target for chemical intervention.

#### 5.2.2. Inhibition of NINJ1 Expression

NINJ1, an essential adhesion molecule in the central nervous system, mediates specific interactions between immune cells and endothelial cells. Its role in the pathological processes of cardiovascular diseases and neuroinflammatory disorders has gained increasing attention. Existing studies have demonstrated that inhibiting NINJ1 expression effectively prevents the adhesion and transendothelial migration of human myeloid leukemia cells and human umbilical vein endothelial cells (HUVECs) stimulated by TNF-α, and significantly reduces the migration and adhesion functions of monocytes [105]. Building on this foundation, Toma and colleagues systematically explored the regulatory mechanism of NINJ1 in TNF-α-activated human endothelial cells (HECs). Real-time quantitative PCR and Western blotting assays revealed that, under amlodipine (AML) treatment, both gene transcription and protein expression levels of NINJ1 were substantially decreased in the lysates of TNF-α-stimulated HECs. The results suggest that AML may ameliorate the inflammatory stress response of endothelial cells by modulating the NINJ1 expression pathway [89]. Although the precise molecular mechanism of AML’s action remains to be fully understood and warrants further investigation, this finding provides a valuable theoretical basis for targeted therapy in inflammation-driven diseases.

In addition to amlodipine, other small-molecule compounds have also demonstrated regulatory effects on NINJ1. Studies indicate that phaseolin can significantly inhibit cell–matrix adhesion by downregulating NINJ1 expression [106]. In RAW 264.7 mouse macrophages treated with phenyl-β-D-glucopyranoside [107], the decrease in NINJ1 expression ultimately leads to the suppression of cell adhesion functions. Furthermore, in retinal ischemia–reperfusion (RIR), N, N-dimethyl-3β-hydroxy-cholesterylamine (DMHCA) can directly bind to NINJ1 and inhibit its expression, thereby blocking the NF-κB inflammatory pathway, leukocyte recruitment, and cell death. This process reduces neuroinflammation and retinal structural damage in RIR injury [108]. Meanwhile, in a mouse model of IAV infection, genetic ablation of NINJ1 improves survival, alleviates lung injury and excessive inflammation, and does not impair early antiviral immunity. However, a subset of NINJ1-deficient mice exhibits developmental abnormalities, including growth retardation, hydrocephalus, and ataxia, indicating potential safety concerns associated with gene knockout strategies [16]. Therefore, any prospective targeting approach may need to avoid developmental stages or instead employ adult-stage, cell-type-specific interventions, the feasibility of which warrants further evaluation.

## 6. Conclusions

NINJ1 is a member of the nerve injury-induced protein family, initially discovered in the central nervous system, where it promotes axon growth through mediating homophilic adhesion [1]. As research has advanced, NINJ1 has been shown to be closely linked not only to neurological disorders but also to important roles in various pathological processes, such as cancer and cardiovascular diseases. Kayagaki and colleagues’ research first revealed that NINJ1 can mediate plasma membrane rupture (PMR) [3]. This discovery shifted the research focus from NINJ1-associated disease phenotypes to the mechanisms by which pathogen infection activates NINJ1 and to its specific molecular roles in PMR, thereby providing new therapeutic perspectives for understanding how lytic cell death drives disease progression.

At present, multiple hypotheses have been proposed regarding the activation modes of NINJ1 and the molecular mechanisms underlying its mediation of PMR. Ca^2+^ influx [86] and cell swelling [95] are widely discussed activating factors, and various signaling pathways interacting with NINJ1 have also attracted attention. At the execution level of membrane rupture, the Degen and David groups have proposed membrane-cutting models based on the NINJ1 structure and its interaction with membranes [73,80]. Subsequent studies further demonstrated that increased NINJ1 expression lowers the threshold for mechanically induced PMR, suggesting a cooperative role of mechanical stress [84]. Notably, unpublished data from the Ramos group indicate that commonly used human cell lines can release LDH despite limited endogenous NINJ1 levels, implying that additional proteins may participate in plasma membrane rupture in human cells [27]. However, these perspectives are largely focused on molecular mechanisms, and the role of NINJ1 in the context of pathogen infection remains insufficiently explored. Key unresolved questions include whether different classes of pathogens (viruses, bacteria, fungi) activate NINJ1 through conserved or distinct pathways; whether NINJ1 serves as a common downstream executor of pathogen-induced cell lysis; and whether certain pathogens, having evolved mechanisms to inhibit host cell apoptosis [109], can specifically suppress or enhance NINJ1 oligomerization and membrane-disrupting activity. Elucidating the interactions between pathogens and NINJ1 will not only advance our understanding of infection pathogenesis but may also uncover novel therapeutic targets for anti-infective strategies.

In NINJ1-dependent cell death, the release of large amounts of DAMPs (e.g., HMGB1, histones) following PMR [3] can activate inflammatory responses. While a moderate inflammatory response is essential for pathogen clearance, excessive inflammatory storms can cause severe tissue damage. Therefore, precise regulation of NINJ1 activity during infection—balancing pathogen elimination with the prevention of immune-mediated injury—and defining the boundary between its “beneficial” and “harmful” effects are critical for developing therapeutic strategies that harness the dual nature of NINJ1. Currently reported NINJ1-targeting agents (e.g., glycine, muscimol) can inhibit NINJ1 activity but exhibit limitations such as reversibility and relatively high effective concentrations [92], prompting consideration of upstream intervention strategies such as gene therapy. For example, miRNA-based approaches (e.g., miR-125a-5p) have provided new avenues for the treatment of inflammatory diseases [110].

In summary, future research should aim to elucidate the precise activation mechanisms and upstream regulatory nodes of NINJ1, enable rational drug design based on the structural basis of its membrane-disrupting function, and develop selective, context-dependent strategies to modulate its activity.

## Figures and Tables

**Table 1:** Types of cell death induced by several common pathogens.

Pathogen	Target Cell Types	Cell Death Modalities (Induced/Inhibited)	Effect on Pathogen Fitness	Host Pathological Outcome	References
IAV	Respiratory epithelial cells, Macrophages	Induced: Necroptosis, Pyroptosis, Apoptosis, PANoptosis	Enhanced replication and dissemination; PANoptosis amplifies progeny release	Cytokine storm, acute lung injury, ARDS	[7,8,9,10,11,12]
SARS-CoV-2	Respiratory epithelial cells, Endothelial cells, Monocytes/Macrophages	Induced: Necroptosis, Pyroptosis, PANoptosis	Early suppression of apoptosis facilitates viral replication; later induction of necroptosis/pyroptosis promotes inflammation-driven spread	Cytokine storm, endothelial damage, multi-organ failure	[13,14,15,16]
MERS-CoV	Respiratory epithelial cells	Induced: Intrinsic Apoptosis, Extrinsic Apoptosis	Enhanced viral pathogenicity through dual apoptosis pathway activation	Severe alveolar damage, pulmonary fibrosis	[17]
HIV-1	CD4^+^ T lymphocytes, Macrophages	Induced: Apoptosis (both direct via viral proteins Vpr/Tat/Nef and indirect via bystander effects)	Immune cell depletion facilitates secondary infections; enhances pathogenicity of co-infecting pathogens	Progressive immune deficiency (AIDS)	[18,19]
Ebola Virus	Monocytes, Macrophages, Dendritic cells	Induced: Pyroptosis, Necroptosis (both direct and bystander-mediated)	Enhanced viral pathogenicity and systemic dissemination	Hemorrhagic fever, multi-organ failure	[20]
*Staphylococcus aureus*	Phagocytes (Macrophages, Neutrophils), Epithelial cells	Inhibited (early): Apoptosis, Pyroptosis Induced (late): Pyroptosis, Necroptosis, Necrosis	Early immune evasion enables intracellular survival Late-stage lytic escape facilitates bacterial dissemination to new host cells	Tissue abscess formation, bacteremia, sepsis	[21]
*Mycobacterium tuberculosis*	Alveolar Macrophages	Inhibited: Apoptosis Induced: Programmed Necrosis	Suppression of apoptosis allows stable intracellular replication niche Necrosis facilitates bacterial release and transmission	Granuloma formation, caseous necrosis, cavitary lesions	[22]
*Yersinia* spp.	Macrophages, Dendritic cells	Inhibited: Apoptosis, Pyroptosis (via YopJ/YopM/YopK effector proteins)	Sustained inhibition of host defense pathways enables persistent infection	Lymphadenitis, systemic dissemination	[23]

## Data Availability

No new data were created or analyzed in this study. Data sharing is not applicable to this article.
